# La^3+^ Networks and Speciation in the Molten State: Impact of Spacer Salt Selection on Structural Heterogeneity

**DOI:** 10.1021/jacs.5c22776

**Published:** 2026-03-19

**Authors:** Bichitra Borah, Matthew S. Emerson, Santanu Roy, John J. Ferrari, Karena W. Chapman, Leighanne C. Gallington, Diwash Dhakal, Ellie M. Kim, Phillip W. Halstenberg, Sheng Dai, Simerjeet K. Gill, James F. Wishart, Claudio J. Margulis

**Affiliations:** † Department of Chemistry, 4083 The University of Iowa, Iowa City, Iowa 52242, United States; ‡ Chemistry Department, 8099 Brookhaven National Laboratory, Upton, New York 11973, United States; § Chemical Sciences Division, 6146 Oak Ridge National Laboratory, Oak Ridge, Tennessee 37831, United States; ∥ Department of Chemistry, 12301 Stony Brook University, 100 Nicolls Road, Stony Brook, New York 11790, United States; ⊥ X-ray Science Division, Advanced Photon Source, 1291 Argonne National Laboratory, Argonne, Illinois 60439, United States; # Nuclear Science and Security Department, Brookhaven National Laboratory, Upton, New York 11973, United States; ∇ Department of Chemistry, 4285 University of Tennessee Knoxville, Knoxville, Tennessee 37996, United States; ○ Chemical Sciences Division, Oak Ridge National Laboratory, Oak Ridge, Tennessee 37831, United States

## Abstract

We recently introduced the concept of a “spacer salt” that creates structural heterogeneity and intermediate range order. Put simply, a fully networked salt melt, such as LaCl_3_ or UCl_3_, becomes disrupted by the introduction of ions that do not participate in the network. One of the results of this disruption is the experimental observation of two characteristic distances between the multivalent cations: the shorter “in-network” distance and the longer “across-network” distance spaced by the low-valency salt. The longer characteristic distance, absent if there is no spacer salt, is the culprit for a new first sharp diffraction peak in scattering experiments. Intuitively, it would appear to follow from this analysis that higher concentrations of the lower-valency salt would further separate multivalent cations, resulting in a shift to lower *q* values of this first sharp diffraction peak. We will show experimentally and computationally that this is not always the case because multiple other factors enter into play.

## Graphical abstract

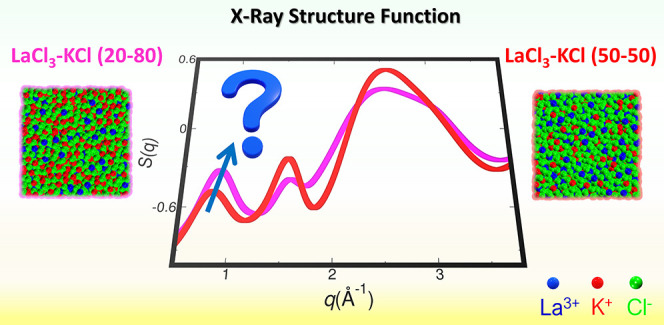

Molten salts have tremendous potential^1^ for advanced energy applications including as fuel or coolant in next-generation modular molten salt reactor (MSR) technologies.^2–5^ Salts of the actinides and lanthanides are particularly important in one case as fuel material and in the other as elements that accumulate as fission products. Separation of lanthanides and actinides using high-temperature molten salts is also a very exciting area of research.^6,7^ In this context, understanding the structural landscape of complex mixture melts including actinide and lanthanide cations is fundamentally important,^8–12^ but synchrotron experiments on high-temperature molten salts remain a significant challenge. For LaCl_3_–NaCl and LaCl_3_–KCl mixture melts at 20 mol % and 50 mol % in LaCl_3_ (see description of sample preparation in Section S.1.1), Figure 1a,b shows how remarkably good the agreement between our recently measured and simulated *S*(*q*) functions is, giving confidence in the interpretation of results based on our polarizable ion model (PIM) molecular dynamics (MD) simulations (see Sections S.1 and S.2 for full experimental and computational protocols).

**Figure 1:**
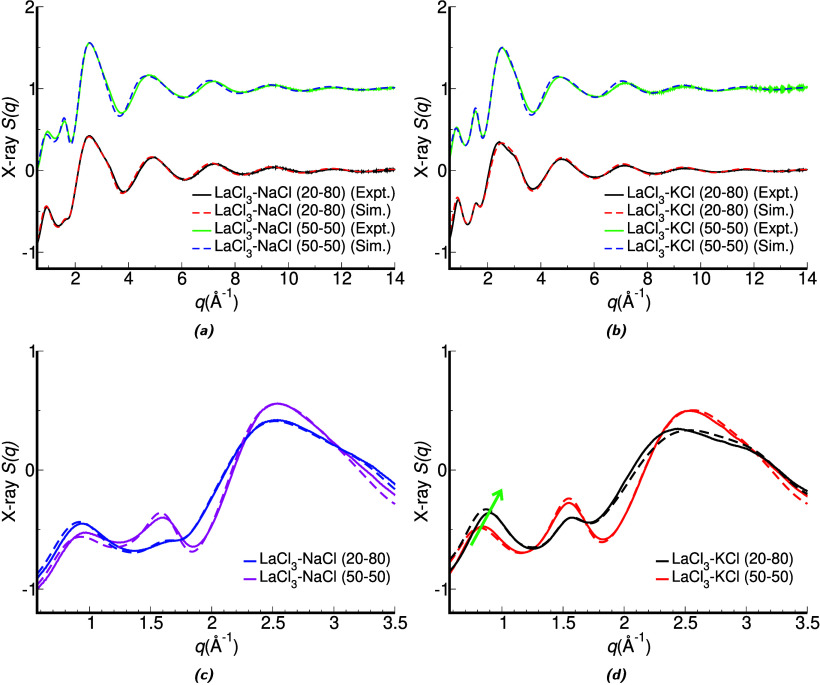
(Top) Experimental and simulated X-ray *S*(*q*) for 50:50 and 20:80 mol % LaCl_3_–KCl and LaCl_3_–NaCl melts at 1173 K; 50% compositions shifted by +1. (Bottom) Unshifted, zoomed in views emphasizing *q* regions associated with the spacer-salt prepeak (*q* < 1 Å^–1^), in-network charge alternation (*q* ∼ 1.5 Å^–1^), and higher *q* adjacency correlations.

In ref 9 we proposed that when LaCl_3_ is mixed with NaCl, a new first sharp diffraction peak (also called a prepeak) appears at low *q* values indicative of intermediate range order which (i) was absent in the case of the neat LaCl_3_ melt and which (ii) follows anti-Debye–Waller behavior (its intensity increases with increasing temperature). In prior work we have demonstrated that such allegedly anomalous behavior is in fact normal behavior for prepeaks in ionic liquids and molten salts.^9,13–18^ Reference 9 explained this new prepeak, absent in neat LaCl_3_ or NaCl,^9,19^ as resulting from a “spacer salt effect”. This is because the prepeak appears due to a new characteristic distance between multivalent cations that do not share counterions and are separated by the lower valency salt. Later we found that the spacer salt effect is not unique to LaCl_3_ in mixtures with NaCl but is, instead, more general. For example, the same type of prepeak appears in mixtures of UCl_3_ with KCl that was absent for neat UCl_3_.^10^

Such a simple and satisfying idea that spacer-salt prepeaks appear in mixture melts because the monovalent spacer salt separates the percolating anion-decorated network of the actinide or lanthanide cations could easily lead to the following hypothesis: a larger concentration of spacers must result in (1) increasingly larger separation of multivalent metal ions and (2) a prepeak that increasingly shifts to lower *q* values.

Figures 1c and 1d clearly debunk that hypothesis; experiments and simulations show that for mixtures of LaCl_3_ with NaCl (Figure 1c) the prepeak at or below 1 Å^–1^ hardly moves to lower *q* values with an increase of the spacer salt concentration, and in the case of mixtures of LaCl_3_ with KCl (Figure 1d) the prepeak moves significantly but in the “wrong” direction (see green arrow guiding the eye). Figure S1 shows that the prepeak shift to higher *q* values in the case of dilution with KCl is also observed in the simulated neutron *S*(*q*), ruling out electronic effects (the *q*-dependence of the X-ray form factors) as a contributing factor.

Whereas this article focuses on the two aforementioned compositions, Figure S2 demonstrates that the observed anomalous trend in the case of KCl holds true for a variety of melt concentrations. What this means is that dilution does more to the architecture of Cl^–^-decorated La^3+^ ion charge networks than simply separating them, and Figure 1 demonstrates that it is not trivial to predict how increased dilution will affect the position of the prepeak.

Evidence of changes in the architecture of the Cl^–^-decorated La^3+^ charge network upon dilution of LaCl_3_ from 50% to 20% with KCl can be gleaned from the corresponding La^3+^–Cl^–^ and La^3+^–La^3+^ partial subcomponents of *S*(*q*) depicted in Figure S3 (left). We see from Figure S3 (left) that, just as in Figure 1d, partial subcomponent prepeaks below *q* = 1 Å^–1^ shift to higher *q*. Instead, upon dilution, the charge alternation antipeak for La^3+^–Cl^–^ (negative going peak between 1.5 and 2 Å^–1^ in Figure S3 (left)) shifts to lower *q*; the changes are highlighted with green arrows in Figure S3 (left) to guide the eye. Notice also that consistent with Figure 1c, no significant shifts are observed for LaCl_3_–NaCl in the prepeak region of Figure S3 (right) or in the charge alternation antipeak for La^3+^–Cl^–^ upon dilution.

A quantitative view of real-space structural changes occurring in each mixture melt upon dilution can be derived from cluster or network size analysis.^20–22^ Figure 2 shows the probability distribution of La^3+^ cluster sizes defined for a La^3+^–La^3+^ cutoff value of 6.4 Å matching the minimum of the first peak in the La^3+^–La^3+^ pair distribution function (*g*(*r*)) shown in Figure S5. Notice from Figure S5 that the first minimum and also the first maximum are remarkably consistent across mixture melts and concentrations, making the cluster analysis across systems easily comparable. Data shown in Figure 2 (right) indicate that when the LaCl_3_ concentration is 50%, both the LaCl_3_–KCl and the LaCl_3_–NaCl mixture melts are highly networked; the probability of Cl^–^-decorated La^3+^ networks with hundreds of metal ions (in our simulations this means a percolating network) is predominant. As expected for mixture melts with NaCl or KCl, lowering the concentration of La^3+^ cations also decreases the level of multivalent metal ion connectivity (the size of the aggregates). However, Figure 2 (left) shows that when contrasted with the case of KCl, the mixture melt with NaCl preserves a much larger fraction of large networks upon dilution, and there are fewer Cl^–^-decorated single La^3+^ ion “complexes”. In other words, each spacer salt has a distinct effect on the multivalent metal ion network architecture. Figure S4 shows typical simulation snapshots that pictorially exemplify the results presented in Figure 2. Dilute LaCl_3_ in KCl appears to show fewer network connections (orange links) compared to dilute LaCl_3_ in NaCl where we still notice complex La^3+^ ion network connectivities.

**Figure 2:**
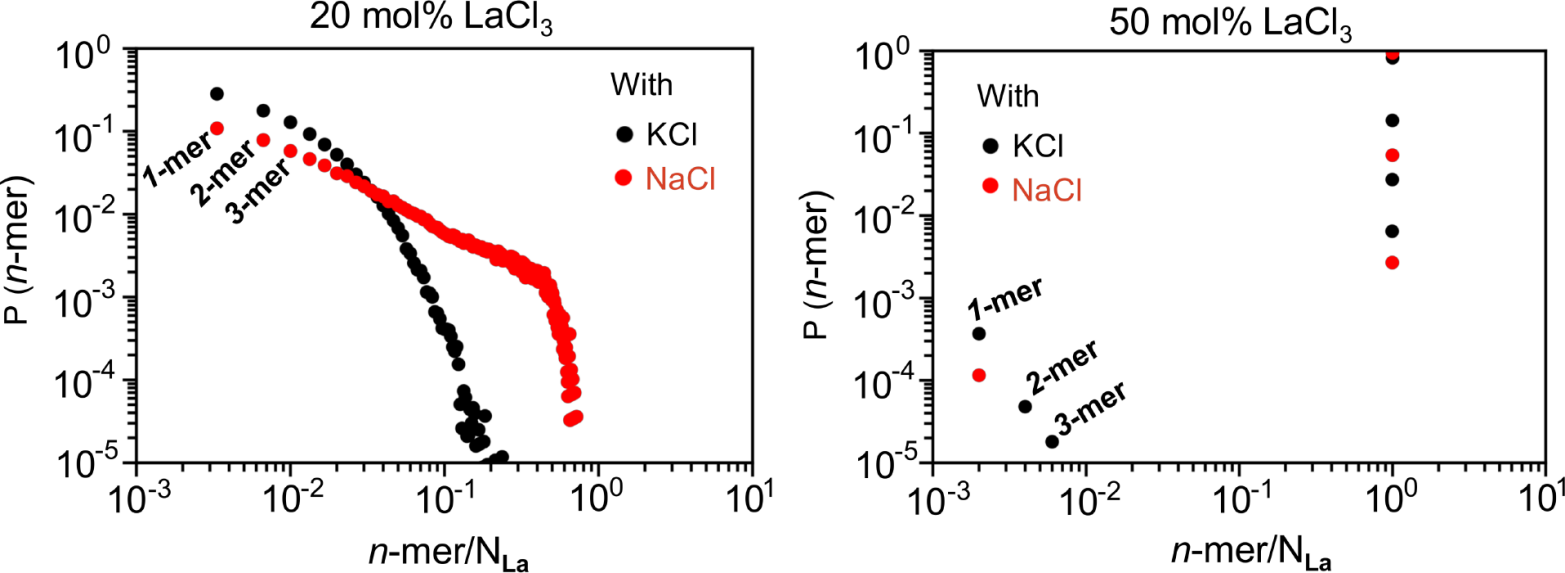
Probability of a La^3+^ aggregate or network as a function of the aggregate size defined as the number of La^3+^ ions in the aggregate divided by the total number in the simulation box.^22^

A deeper dive into subtle but important structural differences across melts can be accomplished by exploring free energies in the condensed phase.^8,9,18^ ^,^ ^23–25^ Figure 3 shows free energy functions (*W*(*r*, *CN*)) where *r* (the X coordinate in each graph) is always the distance between pairs of La^3+^ metal ions in the melt, and *CN* (the Y coordinate) is either (i) the number of Cl^–^ ions coordinating with any of the La^3+^ metal ions in the pair or (ii) the number of neighboring La^3+^ ions to any of the La^3+^ ions in the pair. In each case, neighboring coordination considers ions up to the first minimum of the relevant *g*(*r*) and is defined via a continuous function (see Figures S5, S6, and Section S.2.2). This makes definitions used for the free energy and cluster analysis consistent.

**Figure 3:**
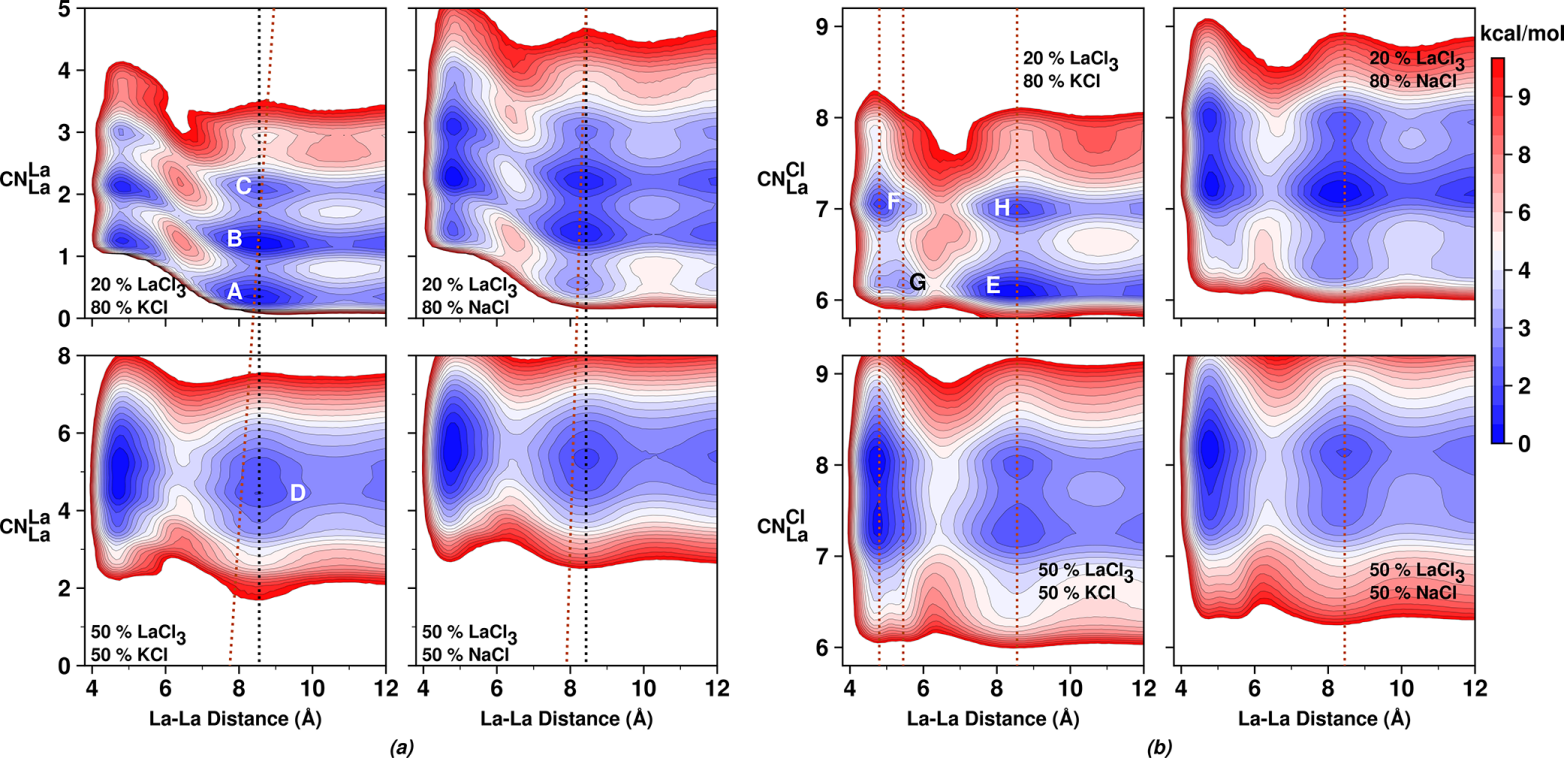
For mixture melts at different concentrations. 2D free-energy landscapes in which the X axis is always the distance between a reference pair of La^3+^ ions and the Y axis is (a) the La^3+^ coordination of either of these La^3+^ ions, (b) the Cl^–^ coordination of either of these La^3+^ ions. See Section S.2.2 for functional definitions and cutoff values. Straight lines guide the eye.

Consider first Figure 3a in which each of the four subfigures shows a minimum or set of minima around X ∼ 5 Å. These specific free energy minima represent types of in-network connections between La^3+^ ions that share counterions (think of these free energy minima as related to the peaks and antipeaks in the charge alternation regime of *S*(*q*)). Subfigures within Figure 3b also show a set of minima at X ∼ 5 Å; in this case, we learn about the typical Cl^–^ ion coordination around either La^3+^ ion of the reference pair when these are adjacent (in-network). For instance, minima labeled F and G are commonly representative of edge-shared and corner-shared configurations, respectively (as an example, see ref 9).

The shrinkage in the La^3+^ ion cluster size depicted in Figure 2, or as a proxy for it, the significant loss in connectivity between multivalent metal ions upon dilution, can be clearly gleaned from Figure 3a. For example, at a concentration of 50% in LaCl_3_, the distribution of La^3+^ ions neighboring a member of the pair when these two are ∼ 5 Å apart is centered at ∼ 5 or 6 depending on the mixture melt; in each melt, the value shifts to below ∼ 4 when the concentration of LaCl_3_ is 20%.

The previous analysis of free energy minima at X ∼ 5 Å, which is consistent with prior EXAFS results,^11^ offers much insight into the topology of the charge network, and its complex connectivity. However, the prepeak, intermediate range order, and the spacer salt effect are related instead to spatial correlations happening at La^3+^–La^3+^ distances longer than ∼ 5 Å. Because of this, we shift our focus to the next set of free energy minima that can be found in the regime X ∼ 8 Å, think of multivalent metal ions that are relatively close, but do not share Cl^–^ counterions; in other words, typical “across network or across complexes behavior”, as opposed to the “within network behavior” case described for X ∼ 5 Å.

As an example, consider minimum A, which represents the case of two La^3+^ ions at a distance of X ∼ 8 Å when at least one of them is not part of a network (Y ∼ 0). *Put differently, minimum* A *occurs when a* Cl^–^ *-decorated* La^3+^ *ion is across a* La^3+^ *complex instead of a* La^3+^ *that is part of a network*. Notice how minimum A is highly popular at 80% KCl, but the analogous Y ∼ 0 minimum in the 80% NaCl melt is shallow; this is consistent with the cluster analysis in Figure 2(left) where the mixture with KCl shows significantly more monomers (metal complexes instead of networks) compared to the case of the melt containing NaCl at the same concentration. This is also consistent with the qualitative network depictions in Figure S4.

Interestingly, and perhaps related to the allegedly “wrong” direction in which the prepeak shifts when diluting with KCl the 50% LaCl_3_ mixture melt, we notice that correlations in the X ∼ 8 Å regime become shorter (smaller value of X, larger value of *q*) when networks are smaller. To see this, compare the X value of minima C, B, and A. In other words, an increase in the population of multivalent metal ion complexes, as opposed to metal ion networks, or an increase in the population of smaller La^3+^ ion aggregates should shift the spacer salt prepeak to higher *q*, and these smaller aggregates appear more prominently for dilute KCl.

If we consider the changes in Cl^–^ ion coordination upon dilution for across network (or across complex) La^3+^ ions, those in the range X ∼ 8 Å, we find that for the 50% LaCl_3_–NaCl melt, *CN* 8 is dominant with some population at a value of 7. These two are also the most popular coordination numbers for the 50% LaCl_3_–KCl melt. Diluting the LaCl_3_–NaCl melt with NaCl preserves 8 and 7 as the most prominent *CN* values, but we also observe some population at a value of 6; instead, diluting the LaCl_3_–KCl melt results in a new and very prominent *CN* ∼ 6 (minimum E).

In conclusion, mixing LaCl_3_ with monovalent salts results in a spacer salt prepeak. However, it is not simple to predict how this prepeak will shift with changes in concentration of the spacer. This is because the prepeak depends on the statistics of multivalent metal ion aggregate spacing and coordination which in turn depend in a nontrivial way on the identity of the spacer.

## Supplementary Material



## Data Availability

Data sets for this article are made available within 30 days of the official acceptance date of this article by the journal in the Zenodo repository under the Digital Object Identifier (DOI): 10.5281/zenodo.19006532.

## Notes

Experimental Section including sample preparation and scattering measurements, Simulation Section including a description of the polarizable ion model, equilibration protocols, production runs, the computation of *S*(*q*) for neutron and X-ray scattering, partial subcomponents of *S*(*q*), definition of coordination numbers used in the computation of free energies, Additional Figures including: computational neutron *S*(*q*), computational X-ray *S*(*q*) for extra intermediate concentrations, selected partial subcomponents of X-ray *S*(*q*), simulation snapshots for 20:80 mol % mixture melts showing La^3+^ ion connectivities, *g*(*r*) functions for La^3+^–La^3+^ and for La^3+^–Cl^–^ (PDF)

## References

[1] Roper R., Harkema M., Sabharwall P., Riddle C., Chisholm B., Day B., Marotta P. (2022). Molten salt
for advanced energy applications: A review. Ann. Nucl. Energy.

[2] Rosenthal M. W., Kasten P. R., Briggs R. B. (1970). Molten-Salt Reactors—History,
Status, and Potential. Nucl. Appl. Technol..

[3] LeBlanc D. (2010). Molten salt
reactors: A new beginning for an old idea. Nucl.
Eng. Des..

[4] Walker, S. A. ; Tano, M. E. ; Abou-Jaoude, A. ; Calvin, O. Depletion-driven thermochemistry of molten salt reactors: review, method, and analysis. Front. Nucl. Eng. 2023, 2. 10.3389/fnuen.2023.1214727

[5] IAEA. Status of Molten Salt Reactor Technology. Technical Reports Series No. 489; International Atomic Energy Agency, 2023

[6] Yin T.-q., Xue Y., Yan Y.-d., Ma Z.-c., Ma F.-q., Zhang M.-l., Wang G.-l., Qiu M. (2021). Recovery and separation of rare earth
elements by molten salt electrolysis. Int. J.
Miner. Metall. Mater..

[7] Galashev A. Y. (2022). Recovery
of actinides and fission products from spent nuclear fuel via electrolytic
reduction: Thematic overview. Int. J. Energy
Res..

[8] Emerson M.
S., Ivanov A. S., Gallington L. C., Maltsev D. S., Halstenberg P., Dai S., Roy S., Bryantsev V. S., Margulis C. J. (2024). Heterogeneous Structure,
Mechanisms of Counterion Exchange, and the Spacer Salt Effect in Complex
Molten Salt Mixtures Including LaCl_3_. J. Phys. Chem. B.

[9] Emerson M. S., Sharma S., Roy S., Bryantsev V. S., Ivanov A. S., Gakhar R., Woods M. E., Gallington L. C., Dai S., Maltsev D. S., Margulis C. J. (2022). Complete Description of the LaCl_3_–NaCl Melt Structure and the Concept of a Spacer Salt
That Causes Structural Heterogeneity. J. Am.
Chem. Soc..

[10] Emerson M. S., Ogbodo R., Margulis C. J. (2024). Spiers Memorial Lecture: From cold
to hot, the structure and structural dynamics of dense ionic fluids. Faraday Discuss..

[11] Okamoto Y., Suzuki S., Shiwaku H., Ikeda-Ohno A., Yaita T., Madden P. A. (2010). Local Coordination about La3+ in
Molten LaCl3 and Its Mixtures with Alkali Chlorides. J. Phys. Chem. A.

[12] Smith A. (2022). Structure-property
relationships in actinide containing molten salts – A review:
Understanding and modelling the chemistry of nuclear fuel salts. J. Mol. Liq..

[13] Sharma S., Emerson M. S., Wu F., Wang H., Maginn E. J., Margulis C. J. (2020). SEM-Drude Model for the Accurate and Efficient Simulation
of MgCl_2_-KCl Mixtures in the Condensed Phase. J. Phys. Chem. A.

[14] Santos C. S., Annapureddy H. V. R., Murthy N. S., Kashyap H. K., Castner E. W., Margulis C. J. (2011). Temperature-dependent
structure of
methyltributylammonium bis­(trifluoromethylsulfonyl)­amide: X ray scattering
and simulations. J. Chem. Phys..

[15] Hettige J. J., Kashyap H. K., Margulis C. J. (2014). Communication:
Anomalous temperature
dependence of the intermediate range order in phosphonium ionic liquids. J. Chem. Phys..

[16] Araque J.
C., Hettige J. J., Margulis C. J. (2015). Modern Room Temperature Ionic Liquids,
a Simple Guide to Understanding Their Structure and How It May Relate
to Dynamics. J. Phys. Chem. B.

[17] Borah B., Acharya G. R., Grajeda D., Emerson M. S., Harris M. A., Milinda Abeykoon A., Sangoro J., Baker G. A., Nieuwkoop A. J., Margulis C. J. (2023). Do Ionic Liquids Slow Down in Stages?. J. Am. Chem. Soc..

[18] Wu F., Sharma S., Roy S., Halstenberg P., Gallington L. C., Mahurin S. M., Dai S., Bryantsev V. S., Ivanov A. S., Margulis C. J. (2020). Temperature Dependence of Short and
Intermediate Range Order in Molten MgCl_2_ and Its Mixture
with KCl. J. Phys. Chem. B.

[19] Sharma S., Ivanov A. S., Margulis C. J. (2021). A Brief
Guide to the Structure of
High-Temperature Molten Salts and Key Aspects Making Them Different
from Their Low-Temperature Relatives, the Ionic Liquids. J. Phys. Chem. B.

[20] Shimizu K., Bernardes C. E. S., Canongia Lopes J. N. (2014). Structure
and aggregation in the
1-alkyl-3-methylimidazolium bis­(trifluoromethylsulfonyl)­imide ionic
liquid homologous series. J. Phys. Chem. B.

[21] Bernardes C. E. S., Shimizu K., Lobo Ferreira A. I. M.
C., Santos L. M. N. B. F., Canongia Lopes J. N. (2014). Structure and aggregation in the
1,3-dialkyl-imidazolium bis­(trifluoromethylsulfonyl)­imide ionic liquid
family: 2. From single to double long alkyl side chains. J. Phys. Chem. B.

[22] Bernardes C. E. S. (2017). AGGREGATES:
Finding structures in simulation results of solutions. J. Comput. Chem..

[23] Roy S., Sharma S., Karunaratne W. V., Wu F., Gakhar R., Maltsev D. S., Halstenberg P., Abeykoon M., Gill S. K., Zhang Y., Mahurin S. M., Dai S., Bryantsev V. S., Margulis C. J., Ivanov A. S. (2021). X-ray Scattering Reveals Ion Clustering
of Dilute Chromium Species in Molten Chloride Medium. Chem. Sci..

[24] Roy S., Brehm M., Sharma S., Wu F., Maltsev D. S., Halstenberg P., Gallington L. C., Mahurin S. M., Dai S., Ivanov A. S., Margulis C. J., Bryantsev V. S. (2021). Unraveling
Local Structure of Molten Salts via X-ray Scattering, Raman Spectroscopy,
and *Ab Initio* Molecular Dynamics. J. Phys. Chem. B.

[25] Wu F. (2019). Elucidating Ionic Correlations Beyond Simple Charge
Alternation in
Molten MgCl_2_-KCl Mixtures. J. Phys.
Chem. Lett..

