# Supplementary material for: La3+ Networks and Speciation in the Molten State: Impact of Spacer Salt Selection on Structural Heterogeneity
Source: J Am Chem Soc. 2026 Mar 19;148(12):12476–80. doi: 10.1021/jacs.5c22776 (PMC13047673; doi:10.1021/jacs.5c22776)
Supplement: Supplementary file 1 [file ja5c22776_si_001.pdf]

Supporting Information: La^{3+} Networks and Speciation in the Molten State: Impact of Spacer Salt Selection on Structural Heterogeneity

Bichitra Borah,[†] Matthew S. Emerson,[*,‡] Santanu Roy,[*,¶] John J. Ferrari,[§] Karena W. Chapman,[§] Leighanne C. Gallington,[‖] Diwash Dhakal,[⊥] Ellie M. Kim,[#] Phillip W. Halstenberg,[#,@] Sheng Dai,[#,@] Simerjeet K. Gill,[⊥] James F. Wishart,[‡] and Claudio J. Margulis[*,†]

†*Department of Chemistry, The University of Iowa, Iowa City, IA 52242, United States*

‡*Chemistry Department, Brookhaven National Laboratory, Upton, NY 11973, United States*

¶*Chemical Sciences Division, Oak Ridge National Laboratory, Oak Ridge, TN 37831, United States*

§*Department of Chemistry, Stony Brook University, 100 Nicolls Road, Stony Brook, NY 11790, United States*

‖*X-ray Science Division, Advanced Photon Source, Argonne National Laboratory, Argonne, IL 60439, United States*

⊥*Nuclear Science and Security Department, Brookhaven National Laboratory, Upton, NY 11973, United States*

#*Department of Chemistry, University of Tennessee Knoxville, Knoxville, TN 37996, United States*

@*Chemical Sciences Division, Oak Ridge National Laboratory, Oak Ridge, TN 37831, United States*

E-mail: memerson@bnl.gov; roys@ornl.gov; claudio-margulis@uiowa.edu

Contents

S.1 Experimental Section **S-2**

S.1.1 Sample Preparation Procedure S-2

S.1.2 Synchrotron X-Ray Scattering Measurements S-2

S.2 Simulations Section **S-4**

S.2.1 Polarizable Ion Model (PIM) Simulations and $S(q)$ Computation S-4

S.2.2 Coordination numbers and 2D free energy surfaces S-6

S.3 Additional Figures **S-7**

References **S-10**

S.1 Experimental Section

S.1.1 Sample Preparation Procedure

$LaCl_3$ (AnhydroBeads™, −10 mesh, ≥ 99.99% trace metals basis) and KCl (99.999% trace metals basis) were purchased from Sigma Aldrich and dried under dynamic vacuum at 750 °C and 700 °C, respectively. NaCl (99.99% metals basis) was purchased from Thermo Scientific Chemicals and dried under dynamic vacuum at 700 °C. Following drying, $LaCl_3$-KCl and $LaCl_3$-NaCl mixtures with molar ratios of 20 : 80 and 50 : 50 were prepared. The mixtures were melted under vacuum in fused silica test tubes (10 mm I.D. × 12 mm O.D.), sealed with Swagelok Ultra-Torr vacuum fittings, until no phase separation was observed.

S.1.2 Synchrotron X-Ray Scattering Measurements

High energy x-ray total scattering data ($\lambda = 0.1811$ Å) were collected at beamline 28-ID-2 of the National Synchrotron Light Source II at Brookhaven National Laboratory. Samples were loaded

into quartz capillaries (Charles Supper 1.0 mm I.D., 1.2 mm O.D.), sealed using high temperature epoxy inside an argon glovebox and mounted on a furnace sample environment.[1] Samples were heated to 900°C and X-ray scattering images were collected using an amorphous-silicon-based flat panel detector (Perkin Elmer).[2,3] Sample temperature was monitored and controlled using the feedback from a contactless IR thermometer fixed above the furnace setup. The IR thermometer was calibrated against a thermocouple placed at the same position inside an identical capillary setup. The sample-to-detector distance, detector tilt, and detector distortions were corrected within GSAS-II[2] based on a scattering image collected for NIST-standard CeO_2. Regions of the two-dimensional images, where the sample environment and/or beamstop partially absorbed the scattered x-ray beam were masked before the images were reduced to one-dimensional scattering intensity data using GSAS-II.[4] Scattering intensity data were converted into PDFs, within PDFgetX2, subtracting the contributions of the background from the measured diffraction intensities.[5] Corrections for Compton scattering, sample fluorescence, oblique incidence, and the energy-dependent detector response were applied to obtain the structure function, $S(q)$. Features in the low r part of the pair distribution function $G(r)$ (data not shown) were used as feedback to iteratively optimize the fluorescence correction and normalization of the experimental data.

The experimental x-ray structure function is calculated by using the following equation:

$$S(q) = \frac{I_{coh}(q) - \sum_i x_i f_i^2(q)}{\left[\sum_i x_i f_i(q)\right]^2} \tag{S.1}$$

In Eq. S.1, I_{coh} is the coherent scattering intensity, i, x_i, and f_i represent the atomic species, atomic fraction, and corresponding x-ray form factor, respectively.

S.2 Simulations Section

S.2.1 Polarizable Ion Model (PIM) Simulations and $S(q)$ Computation

The initial configurations of the salt mixtures were generated with the PACKMOL package.[6] The number of ions corresponding to each composition is provided in Table S.1.

Table S.1: Molten-salt simulation boxes and ion counts. Compositions are $LaCl_3$–(NaCl or KCl) mixtures at the indicated mol %.

Box	Composition (mol %)	La^{3+}	Na^+	K^+	Cl^-
1	$LaCl_3$-NaCl (20–80)	300	1200	–	2100
2	$LaCl_3$-NaCl (30–70)	360	840	–	1920
3	$LaCl_3$-NaCl (40–60)	480	720	–	2160
4	$LaCl_3$-NaCl (50–50)	500	500	–	2000
5	$LaCl_3$-KCl (20–80)	300	–	1200	2100
6	$LaCl_3$-KCl (30–70)	360	–	840	1920
7	$LaCl_3$-KCl (40–60)	480	–	720	2160
8	$LaCl_3$-KCl (50–50)	500	–	500	2000

Simulation protocols and equilibration conditions are summarized in Table S.2. All polarizable ion model (PIM) simulations were conducted in the isothermal–isobaric (NPT)[7] ensemble at 1 bar. Temperature and pressure were controlled using Nosé-Hoover[8,9] thermostat and barostat with chain lengths of 5, with relaxation times (τ) as listed in the Table S.2. Force field parameters, including ionic polarizabilities and charges, charge-dipole damping and Born–Mayer–Huggins (BMH) potential terms, were adopted from Salanne *et al.* and Ishii *et al.*[10,11] Simulations were carried out in periodic cubic boxes using a three-dimensional Ewald summation, as implemented in MetalWalls (version 20.05).[12–14] The real- and reciprocal-space tolerances were set to 1.63e-5 and 1.0e-7, respectively, with a real-space cutoff of 22.677 Bohr ($\approx$12 Å). Induced dipoles were computed at each timestep via self-consistent conjugate gradient minimization with a convergence threshold of 1.0e-7. An initial non-polarizable simulation was used to briefly relax the randomized ionic configuration, followed by annealing steps and a final equilibration stage. A more comprehensive discussion of the methodology is provided in our earlier publication.[15]

Table S.2: PIM NPT Equilibration Procedure

Step Name	Temp. (K)	Time	τ_{Step} (fs)	$\tau_{Thermostat}$(fs)	$\tau_{Barostat}$(fs)	Dipoles?
Relax	1173	10 ps	0.25	100	500	X
Anneal1	1273	40 ps	1.0	100	500	✓
Anneal2	1373	40 ps	1.0	100	500	✓
Anneal3	1500	40 ps	1.0	100	500	✓
Anneal4	1640	40 ps	1.0	100	500	✓
Anneal5	1500	40 ps	1.0	100	500	✓
Anneal6	1373	40 ps	1.0	100	500	✓
Anneal7	1273	40 ps	1.0	100	500	✓
Equil	1173	1 ns	1.0	500	2500	✓
Prod	1173	2 ns	1.0	500	2500	✓

Total x-ray and neutron $S(q)$ functions were calculated from PIM production trajectories based on the following equations:

$$\text{X-ray } S(q) = \frac{\rho_0 \sum_i \sum_j \chi_i \chi_j f_i(q) f_j(q) \int_0^\infty 4\pi r^2 \left(g_{ij}(r) - 1\right) \frac{\sin(qr)}{qr} dr}{\left[\sum_i \chi_i f_i(q)\right]^2} \tag{S.2}$$

$$\text{Neutron } S(q) = \frac{\rho_0 \sum_i \sum_j \chi_i \chi_j b_i b_j \int_0^\infty 4\pi r^2 \left(g_{ij}(r) - 1\right) \frac{\sin(qr)}{qr} dr}{\left[\sum_i \chi_i b_i\right]^2} \tag{S.3}$$

where ρ_0 is the average atomic number density of the system, χ_i and χ_j are the mole fractions of ionic species i and j, $f_i(q)$ and $f_j(q)$ are the x-ray atomic form factors for species i and j, $g_{ij}(r)$ is the pair radial distribution function for species i and j, and b_i, b_j are the coherent neutron scattering length of elements i and j respectively. For further analysis, we decompose the total x-ray $S(q)$ into partial subcomponents:

$$S_{ii}(q) = \frac{\rho_0 \chi_i \chi_i f_i(q) f_i(q) \int_0^\infty 4\pi r^2 \left(g_{ii}(r) - 1\right) \frac{\sin(qr)}{qr} dr}{\left[\sum_i \chi_i f_i(q)\right]^2} \tag{S.4}$$

and

$$S_{ij}(q) = 2 * \frac{\rho_0 \chi_i \chi_j f_i(q) f_j(q) \int_0^\infty 4\pi r^2 \left(g_{ij}(r) - 1\right) \frac{\sin(qr)}{qr} dr}{\left[\sum_i \chi_i f_i(q)\right]^2} \tag{S.5}$$

S.2.2 Coordination numbers and 2D free energy surfaces

To determine the local coordination environment of a La^{3+} ion, we compute the number of chlorides within its first coordination shell ($CN_{\mathrm{La}}^{\mathrm{Cl}}$) and the number of lanthanums ($CN_{\mathrm{La}}^{\mathrm{La}}$) in its second coordination shell from MD trajectories. These coordination numbers–$CN_{\mathrm{La}}^{\mathrm{Cl}}$ and $CN_{\mathrm{La}}^{\mathrm{La}}$–are defined using a continuously differentiable function, f, that accounts for smooth traversals of chlorides (or lanthanums) across the boundary of the first (or second) coordination shell. We set the boundary for the first shell using a cutoff La-Cl distance ($r_{\mathrm{Cl}}^{\dagger} = 4.1$ Å) obtained from the location of the first minimum (after the first peak) of the La-Cl radial distribution function. Similarly, the La-La radial distribution function is used to set the boundary of the second shell ($r_{\mathrm{La}}^{\dagger} = 6.4$ Å). Thus, $CN_{\mathrm{La}}^{\mathrm{Cl}}$ and $CN_{\mathrm{La}}^{\mathrm{La}}$ for an ith La^{3+} ion are expressed as:

$$CN_{\mathrm{La}}^{\mathrm{Cl}} = \sum_{j=1}^{N_{\mathrm{Cl}}} \frac{1-(r_j/r_{\mathrm{Cl}}^{\dagger})^{12}}{1-(r_j/r_{\mathrm{Cl}}^{\dagger})^{24}} = \sum_{j=1}^{N_{\mathrm{Cl}}} f_j \tag{S.6}$$

and

$$CN_{\mathrm{La}}^{\mathrm{La}} = \sum_{j\neq i;j=1}^{N_{\mathrm{La}}} \frac{1-(r_j/r_{\mathrm{La}}^{\dagger})^{12}}{1-(r_j/r_{\mathrm{La}}^{\dagger})^{24}} = \sum_{j\neq i;j=1}^{N_{\mathrm{La}}} f_j. \tag{S.7}$$

Here, N_{Cl} and N_{La} are respectively the total numbers of chlorides and lanthanums present in the system and r_j is computed using periodic boundary conditions. We obtain the joint probability ($P(r,CN)$) of finding a chloride (or a lanthanum) at a distance r from a lanthanum and its coordination number $CN_{\mathrm{La}}^{\mathrm{Cl}}$ (or $CN_{\mathrm{La}}^{\mathrm{La}}$), and compute the associated free-energy surfaces in the form, $W(r,CN) = -k_{\mathrm{B}}T ln[P(r,CN)]$. $k_{\mathrm{B}}T$ is the thermal energy at the temperature T and k_{B} is the Boltzmann constant. These surfaces help us to determine the distribution of the first- and second-shell metastable CN states and the free-energy barriers that separate them.

S.3 Additional Figures

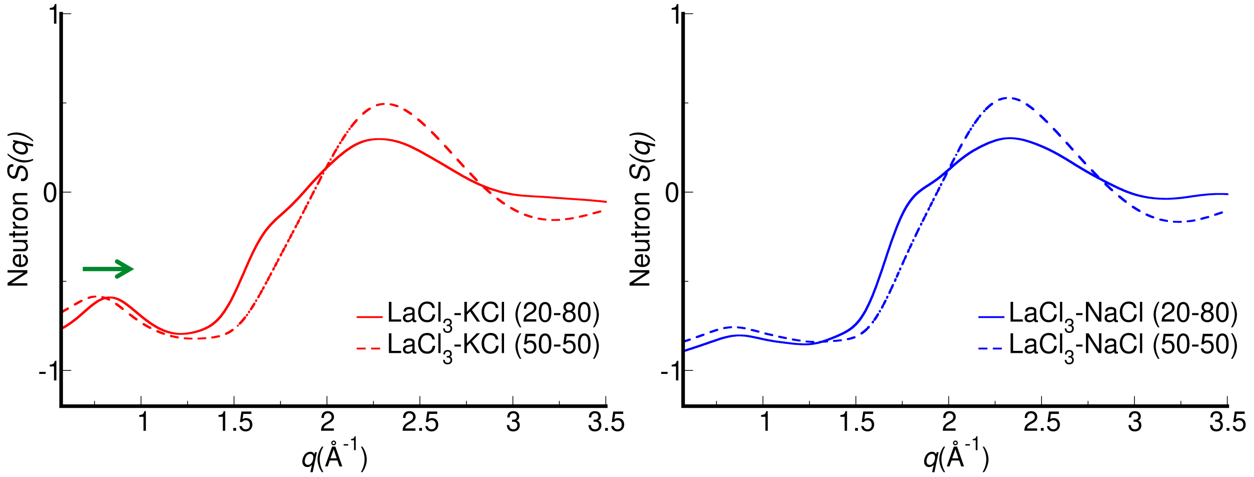

Figure S1: For mixture melts with mol % values shown in each panel, the computational neutron scattering $S(q)$ as defined in Equation S.3. In the case of $LaCl_3$–KCl melts, notice the prepeak shift to higher q values as the La^{3+} ion concentration becomes lower (more spacer salt), and compare this with x-ray scattering results from Figure 1d.

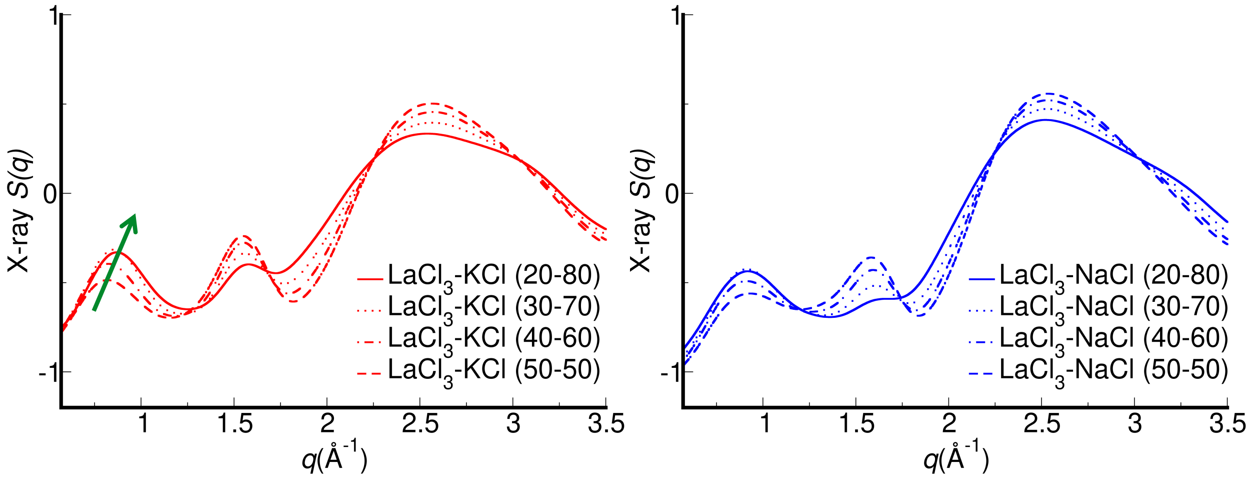

Figure S2: For a larger set of concentrations than those depicted in Figures 1c and 1d, the total computational x-ray $S(q)$ as defined in Equation S.2. Notice the shift of the prepeak to higher q values as the concentration of La^{3+} ion decreases in the case of mixtures with KCl.

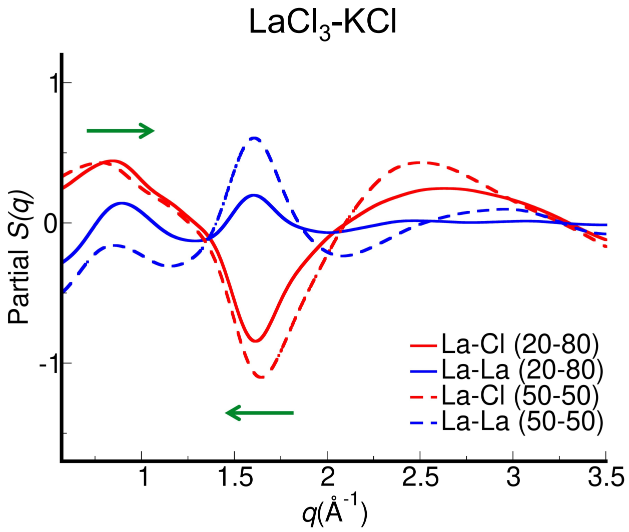

Figure S3: For salt mixtures defined in each panel, partial subcomponents of the computational x-ray $S(q)$ (see Equations S.4 and S.5). Arrows highlight the direction of change as we lower the La^{3+} ion concentration in the case of mixture melts with KCl.

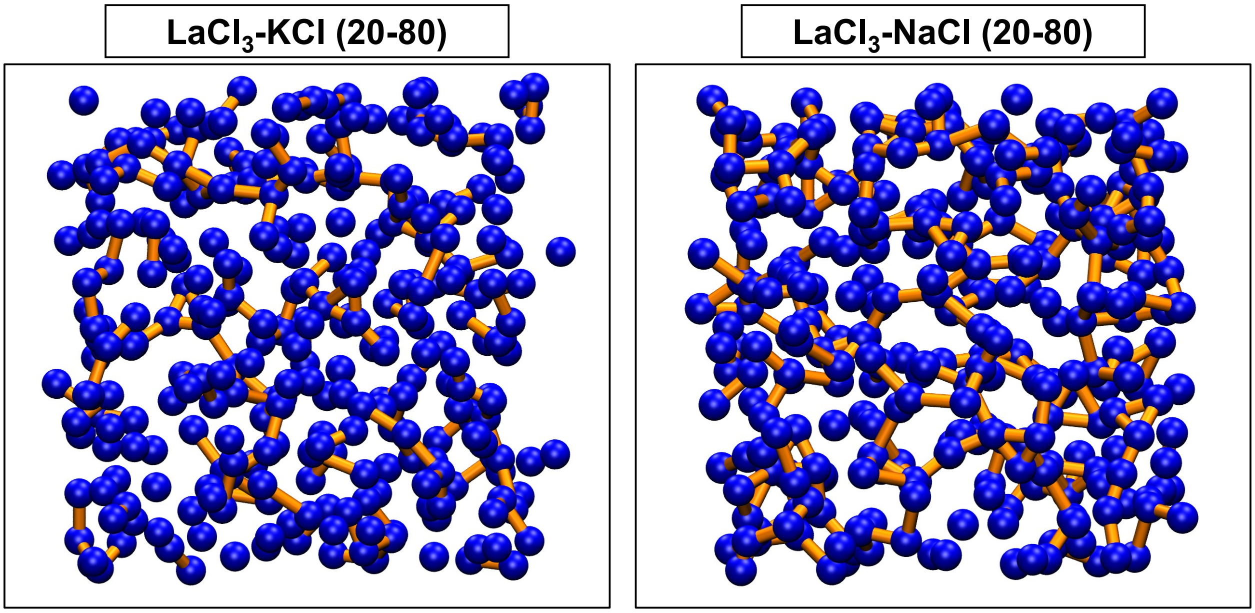

Figure S4: Simulation snapshots at denoted concentrations. Blue spheres are La^{3+} metal ions, other ions not shown. An orange line represents a distance between spheres that is within 6.4 Å. Notice how at this lower concentration of La^{3+}, many of the complex connections still persist in the case of the melt with NaCl.

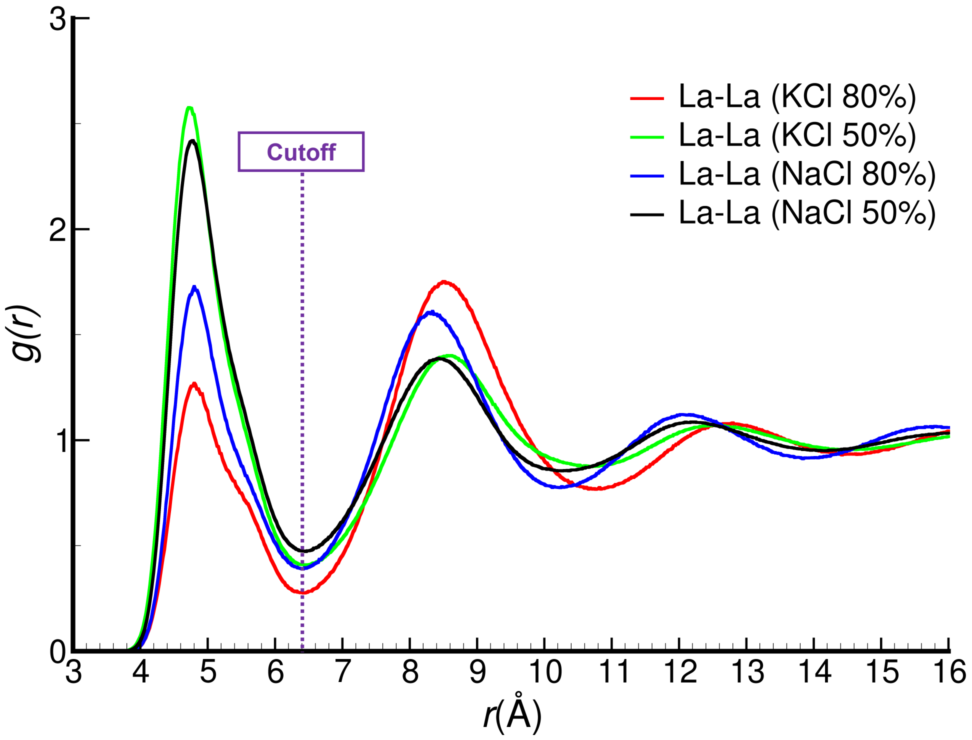

Figure S5: La^{3+}–La^{3+} pair distribution functions showing the cutoff position for aggregate analysis and free energies.

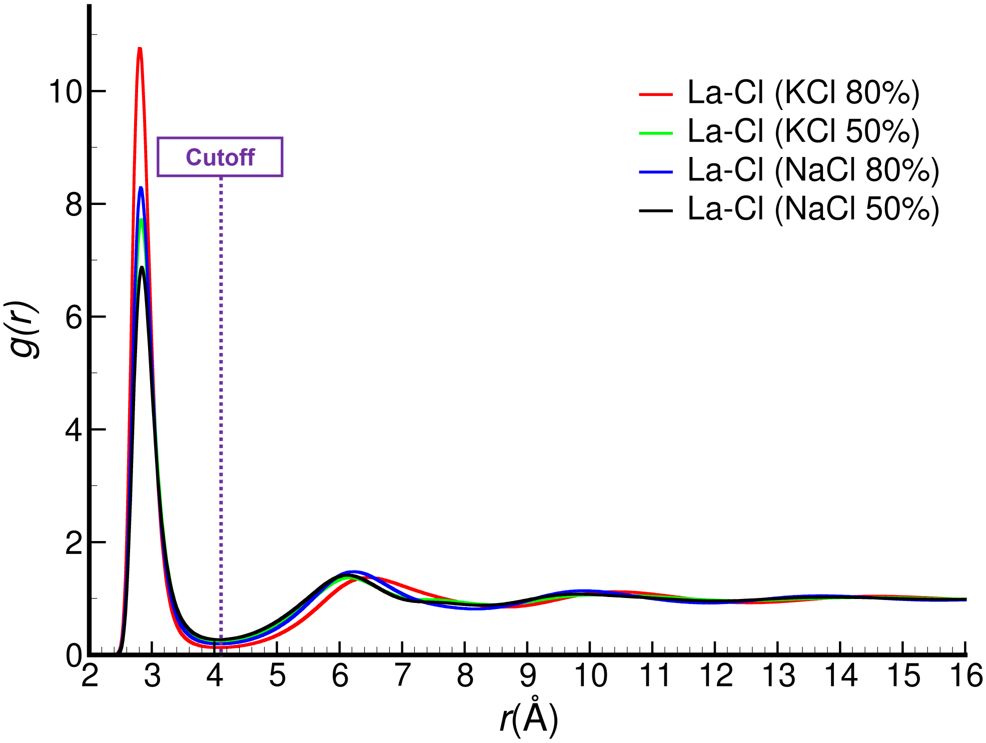

Figure S6: La^{3+}–Cl^{-} pair distribution functions showing the cutoff position for free energy analysis.

References

(1) Chupas, P. J.; Chapman, K. W.; Kurtz, C.; Hanson, J. C.; Lee, P. L.; Grey, C. P. A versatile sample-environment cell for non-ambient X-ray scattering experiments. *J. Appl. Cryst.* **2008**, *41*, 822–824.

(2) Chupas, P. J.; Qiu, X.; Hanson, J. C.; Lee, P. L.; Grey, C. P.; Billinge, S. J. L. Rapid-acquisition pair distribution function (RA-PDF) analysis. *J. Appl. Cryst.* **2003**, *36*, 1342–1347.

(3) Chupas, P. J.; Chapman, K. W.; Lee, P. L. Applications of an amorphous silicon-based area detector for high-resolution, high-sensitivity and fast time-resolved pair distribution function measurements. *J. Appl. Cryst.* **2007**, *40*, 463–470.

(4) Toby, B. H.; Von Dreele, R. B. GSAS-II: the genesis of a modern open-source all purpose crystallography software package. *J. Appl. Cryst.* **2013**, *46*, 544–549.

(5) Qiu, X.; Thompson, J. W.; Billinge, S. J. PDFgetX2: a GUI-driven program to obtain the pair distribution function from X-ray powder diffraction data. *J. Appl. Crystallogr.* **2004**, *37*, 678–678.

(6) Martínez, L.; Andrade, R.; Birgin, E. G.; Martínez, J. M. PACKMOL: A package for building initial configurations for molecular dynamics simulations. *J. Comput. Chem.* **2009**, *30*, 2157–2164.

(7) Martyna, G. J.; Tobias, D. J.; Klein, M. L. Constant pressure molecular dynamics algorithms. *J. Chem. Phys.* **1994**, *101*, 4177–4189.

(8) Nosé, S. A unified formulation of the constant temperature molecular dynamics methods. *J. Chem. Phys.* **1984**, *81*, 511–519.

(9) Hoover, W. G. Canonical dynamics: Equilibrium phase-space distributions. *Phys. Rev. A* **1985**, *31*, 1695–1697.

(10) Salanne, M.; Simon, C.; Turq, P.; Madden, P. A. Calculation of Activities of Ions in Molten Salts with Potential Application to the Pyroprocessing of Nuclear Waste. *J. Phys. Chem. B* **2008**, *112*, 1177–1183.

(11) Ishii, Y.; Kasai, S.; Salanne, M.; Ohtori, N. Transport coefficients and the Stokes–Einstein relation in molten alkali halides with polarisable ion model. *Mol. Phys.* **2015**, *113*, 2442–2450.

(12) Ewald, P. P. Die Berechnung optischer und elektrostatischer Gitterpotentiale. *Ann. Phys.* **1921**, *369*, 253–287.

(13) Aguado, A.; Madden, P. A. Ewald summation of electrostatic multipole interactions up to the quadrupolar level. *J. Chem. Phys.* **2003**, *119*, 7471–7483.

(14) Marin-Laflèche, A.; Haefele, M.; Scalfi, L.; Coretti, A.; Dufils, T.; Jeanmairet, G.; Reed, S.; Serva, A.; Berthin, R.; Bacon, C.; Bonella, S.; Rotenberg, B.; Madden, P.; Salanne, M. MetalWalls: A classical molecular dynamics software dedicated to the simulation of electrochemical systems. *J. of Open Source Softw.* **2020**, *5*.

(15) Emerson, M. S.; Sharma, S.; Roy, S.; Bryantsev, V. S.; Ivanov, A. S.; Gakhar, R.; Woods, M. E.; Gallington, L. C.; Dai, S.; Maltsev, D. S.; Margulis, C. J. Complete Description of the $LaCl_3$–NaCl Melt Structure and the Concept of a Spacer Salt That Causes Structural Heterogeneity. *J. Am. Chem. Soc.* **2022**, *144*, 21751–21762.
